# Identifies Immune Feature Genes for Prediction of Chemotherapy Benefit in Cancer

**DOI:** 10.7150/jca.65646

**Published:** 2022-01-01

**Authors:** Yuquan Bai, Chuan Li, Liang Xia, Fanyi Gan, Zhen Zeng, Chuanfen Zhang, Yulan Deng, Yuyang Xu, Chengwu Liu, Senyi Deng, Lunxu Liu

**Affiliations:** Department of Thoracic Surgery and Institute of Thoracic Oncology, West China Hospital, Sichuan University. Chengdu, 610041.

**Keywords:** Chemotherapy benefit, Immune cell infiltration, Network analysis, prediction model

## Abstract

Chemotherapy is still the most fundamental treatment for advanced cancers so far. Previous studies have indicated that immune cell infiltration (ICI) index could serve as a biomarker to predict chemotherapy benefit in breast cancer and colorectal cancer. However, due to different responses of tumor infiltrating immune cells (TIICs) to chemotherapy, the prediction efficiency of ICI index is not fully confirmed by now. In our study, we first extended this conclusion in 7 cancers that high ICI index could certainly indicate chemotherapy benefit (P<0.05). But we also found the fraction of different TIICs and the interaction of TIICs were varies greatly from cancer to cancer. Therefore, we executed correlation and causal network analysis to identify chemotherapy associated immune feature genes, and fortunately identified six co-owned immune feature genes (CD48, GPR65, C3AR1, CD2, CD3E and ARHGAP9) in 10 cancers (BLCA, BRCA, COAD, LUAD, LUSC, OV, PAAD, SKCM, STAD and UCEC). Base on this, we developed a chemotherapy benefit prediction model within six co-owned immune feature genes through random forest classifying (AUC =0.83) in cancers mentioned above, and validated its efficiency in external datasets. In short, our work offers a novel model with a shrinking panel which has the potential to guide optimal chemotherapy in cancer.

## Introduction

With progressing of medical technology, the clinical treatment for cancer has developed into a combination of surgery, chemotherapy, radiotherapy, targeted therapy, and immune therapy 1. Chemotherapy is still the most fundamental treatment for advanced cancers, especially in the situations of no surgery chance or no sensitive mutation for targeted therapy 2. It is suggested that the implement of chemotherapy could significantly delay disease progression in a variety of cancers, such as lung cancer, liver cancer and colorectal cancer 3-5. However, studies also indicate that even for these chemotherapy sensitive cancers, the response of chemotherapy in patients present greater heterogeneity 6. Therefore, identify effective biomarkers which can indicate chemotherapy benefit and give patients optimized treatment has been one of the long-lived issues in clinical cancer care.

The infiltrations of immune cell and their interactions with cancer cells have formed a unique tumor immune microenvironment (TIME) 7. In TIME, the functions of tumor infiltrating immune cells (TIICs) in cancer evolution are totally different, due to its cell type, density and distribution 8, 9. It has been confirmed that TIICs are widely involved in each step of cancer progression 10, including immune escape, metastasis, drug responses and the prognosis of cancer patients 11, 12. The close correlation between immune cell infiltration and chemotherapy outcome has been reported in cancers, including breast cancer and colorectal cancer 13, 14, which suggested that immune cell infiltration (ICI) index could serve as a biomarker to predict chemotherapy benefit. In addition, studies have reported that in non-small-cell lung cancer, chemotherapy can promote anti-cancer immunity by recruiting T and B cells into TIME and change the phenotype of cytotoxic CD8+ T cells and CD8+/CD4+ memory T cells 15. In triple-negative breast cancer (TNBC) and HER2-positive breast cancer, high levels of ICI in patients indicate a higher complete response rate after neoadjuvant chemotherapy 16, 17. In bladder cancer, patients with more infiltration of these five immune cells (Mast cell, Macrophage, Treg, NK cell and CTLs) has been confirmed could earn better prognosis after chemotherapy 18. However, limited by several studies on the correlation between ICI and chemotherapy outcome and different responses of TIICs to chemotherapy, the prediction efficiency of ICI index is not fully confirmed so far.

In this study, we tried to isolate chemotherapy associated immune feature genes in multiple cancers to construct a new chemotherapy benefit prediction model. Firstly, by download the transcriptome data of cancers from TCGA database, we performed ESTIMATE analyzing and demonstrated that ICI index could indicate chemotherapy benefit in multiple cancers, but the composition and interaction of TIICs were diverse among different caners. Next, we employed correlation and causal gene expression network analysis to identify chemotherapy associated immune feature genes and fortunately got six co-owned immune feature genes. Finally, we developed a chemotherapy benefit prediction model through random forest classifying and validated its efficiency in external datasets. In short, our work offers a novel model with a shrinking panel which has the potential to guide optimal chemotherapy in multiple cancers.

## Materials and Method

### Data and sample collection

Transcriptome data and clinical information data of 33 cancers were downloaded from the TCGA (https://portal.gdc.cancer.gov/) databases. The cancer paraffin-embedded specimens of 52 NSCLC patients were obtained from the Department of Pathology, West China Hospital, Sichuan University, these patients had received neoadjuvant chemotherapy before surgery between January 2016 and August 2020. According to Response Evaluation Criteria in Solid Tumors (RECIST), 7 patients were defined as complete response (CR), 23 patients were defined as partial response (PR) and 22 patients were defined as progressive disease (PD) 19.

### Cancer purity estimation

The ESTIMATE algorithm is used to calculate the immune-stromal component ratio in each cancer sample, and displays with three scores: ImmuneScore, StromalScore and ESTIMATEScore 20. In this study, we use ESTIMATE to measure the immune infiltration levels in mutliple cancers. According to the ImmuneScore, all cancer patients can be divided into two groups.

### Survival analysis

The R packages “survival” and “survminer” were used for survival analysis. The survival curves were estimated by using the Kaplan-Meier method, and the log-rank test was used to analyze differences in survival time.

### Evaluation of specific immune cell infiltration

CIBERSORT was used to evaluate the fraction of specific cell types based on transcriptome data of different cancers 21. Monte Carlo sampling was used to derive the P value in CIBERSORT for the deconvolution of each sample. Cancer samples which P value <0.05 were selected for further analysis.

### Immunohistochemistry

Sections were dewaxed and hydrated, then treated with 10 mM citrate buffer (pH 6.0) for 5 min for antigen repair. After being treated with 3% hydrogen peroxide for 10 min to inactivate endogenous enzymes, sections were blocked in 5% BSA for 20 min and then incubated with primary antibody: CCR2 (Abgent Cat# abs128841, Absin) and UCHL1 (Abgent Cat# 66230-1-lg, Proteintech). Sections were incubated with primary antibody at 4°C overnight. After washing in PBS, sections were incubated with secondary antibodies at room temperature for 30 min. After washing in PBS, sections were treated with streptavidin-biotin complex at room temperature for 20 min. After washing in PBS, the sections were visualized with (3, 30-diaminobenzidine, DAB) DAB. Nucleus was stained with hematoxylin. After dehydration, transparentization and fixation were performed and observed with a light microscope.

### Immunohistochemical evaluation

The grading of positive immunohistochemical reactions is based on the combination of staining intensity and the percentage of positive cells. Five images were randomly obtained for each specimen after ×400 magnification. We try to avoid marginal areas to prevent marginal effects from affecting evaluation. Count all cells and the number of positive cells with a micro-measuring grid, and calculate the average proportion of positive cells. First, score according to the intensity of dyeing: 0 if colorless, 1 if light yellow, 2 if light brown, and 3 if dark brown. Then calculate the percentage of positive cells in each specimen and score it (0 = 0%, 1 = 0% ~ 10%, 2 = 10% ~ 50%, 3 = 50% ~ 75%, 4 = >75 %). Finally, multiply the ratio score by the dyeing intensity score to get the final semi-quantitative score, which is divided into 4 levels: -(0,1,2), +(3,4), ++(6,8), +++ (9,12). Tumors with a final score of more than 3 are considered positive by immunohistochemistry. In terms of protein expression, -~+ means low expression, and ++~+++ means high expression.

### Correlation and causal network analysis

In this study, “edgeR” R package 22 was used to gain differential immune feature genes between ICI high and low chemotherapy group in multiple cancers, the analysis criteria was performed according to the P <0.05 and | log2 fold change (FC) | >2. The R package 'STRINGdb' was used to calculate the correlation between these differential immune feature genes 23. By setting the combined score >0.9, gene-pair with correlation relationship were obtained (Table S3). Among them, top 60 chemotherapy associated immune feature genes were determined for following causal network analysis (Table S4).

The Bayesian causal network with a directed acyclic graph could exhibit the dependent and independent relationships between selected variables, and conditional probability distribution was employed to describe the dependence of variables. We used the “bnlearn” R package to construct the Bayesian causal network among determined chemotherapy associated immune feature genes mentioned above 24. Next, we drew the Bayesian causal network of gene pairs within weight >0.5 (Table S5) by Cytoscape (3.7.2) 25. Finally, we selected the top 20 in-degree and top 20 out-degree genes as the hub nodes of each cancer (Table 1) and identified six co-owned immune feature genes in these cancers.

### Model construction and validation

Based on expression of the six co-owned immune feature genes as characteristic, we constructed a random forest classifier model, 70% of the total patients were selected as train set and the rest patients were selected as test set. We drew the learning curve of n_estimators and adjusted the parameters (max_depth, max_features, min_samples_leaf, min_samples_split) through grid search to get the best combination of each parameter. The feature importance of this model was viewed by feature_importances 26. GSE25055 dataset (breast cancer, n=310) and GSE14814 dataset (non-small cell lung cancer, n=131) were used in external validation. The ROC curve showed the predictive performance of the model in the test and validation sets.

### Functional analysis

We calculated the semantic similarity between chemotherapy associated six co-owned immune feature genes in molecular function and cellular component based on the “GOSemSim” R package, and then calculated the geometric mean to obtain the similarity score of each gene. The most significantly enriched Gene Ontology (GO) terms and Kyoto Encyclopedia of Genes and Genomes (KEGG) for feature genes were identified using DAVID (https://david.ncifcrf.gov/).

## Results

### ICI index indicates chemotherapy benefit in multiple cancers

**Figure 1:**
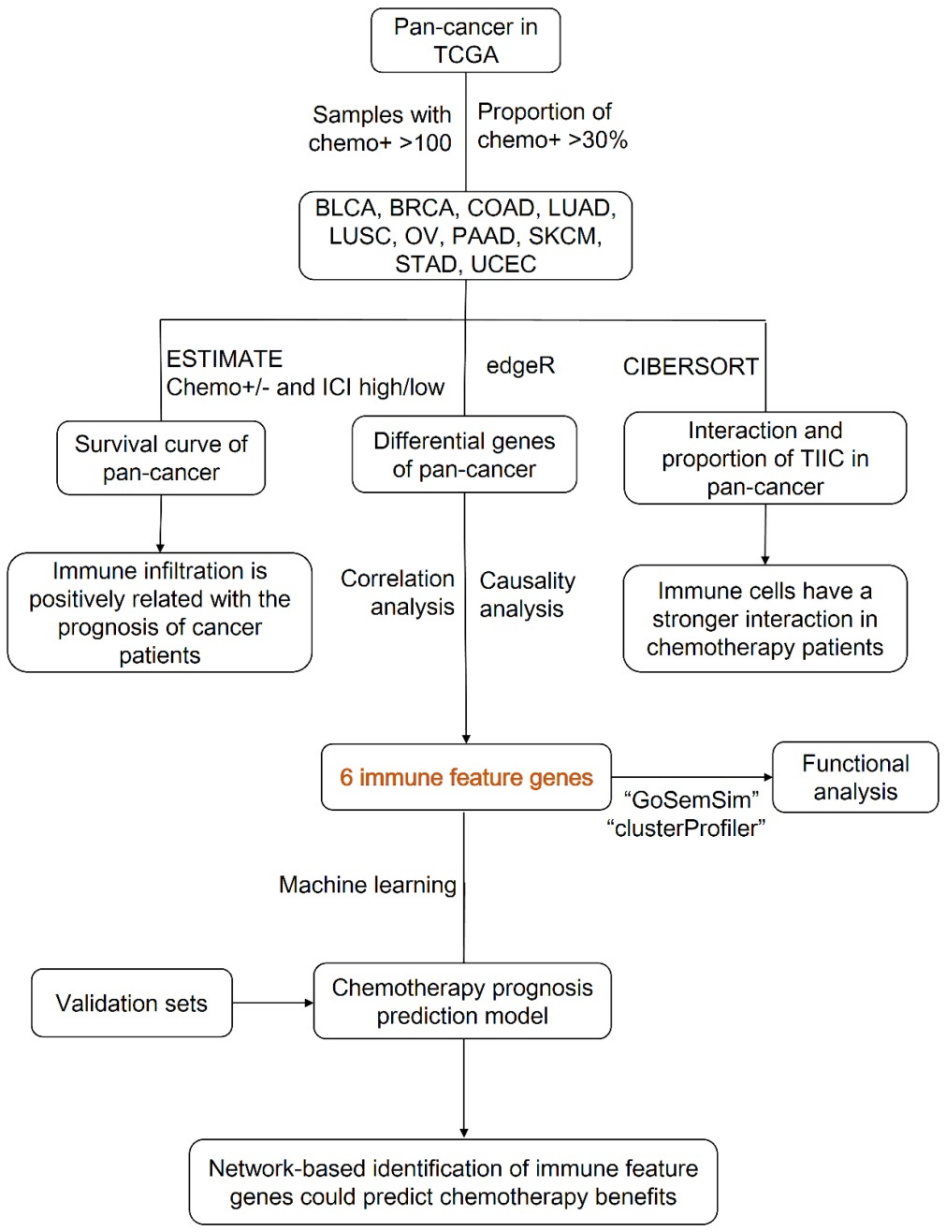
Data screening and analysis process.

**Figure 2:**
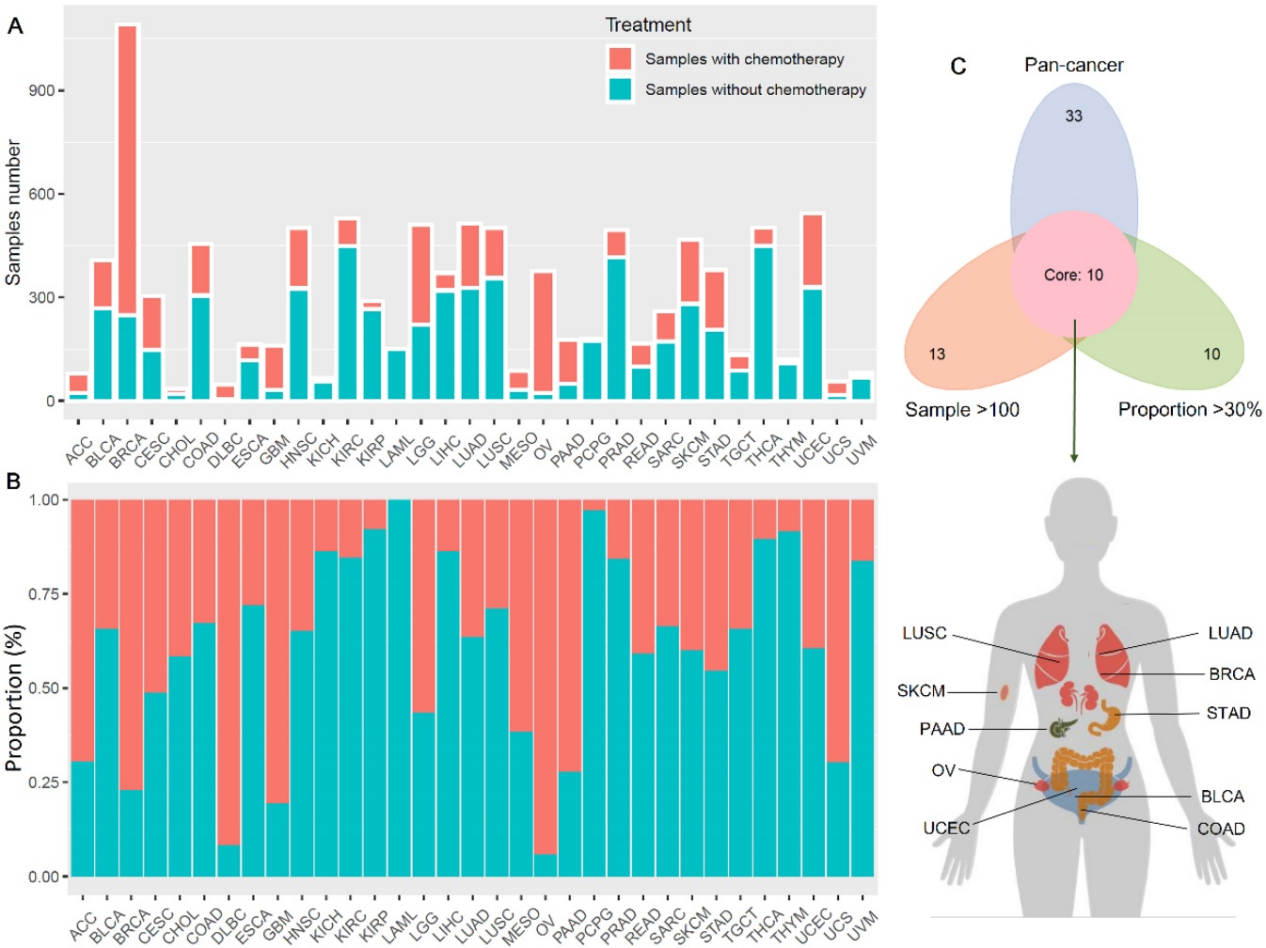
** Chemotherapy information of cancer patients in TCGA.** (**A**) Number of chemotherapy and non-chemotherapy patients in different cancers. (**B**) Percentage of chemotherapy and non-chemotherapy patients in the total patients in different cancers. (**C**) We screened cancers with a proportion of chemotherapy patients >30% and number of chemotherapy patients >100 for subsequent analysis.

**Figure 3:**
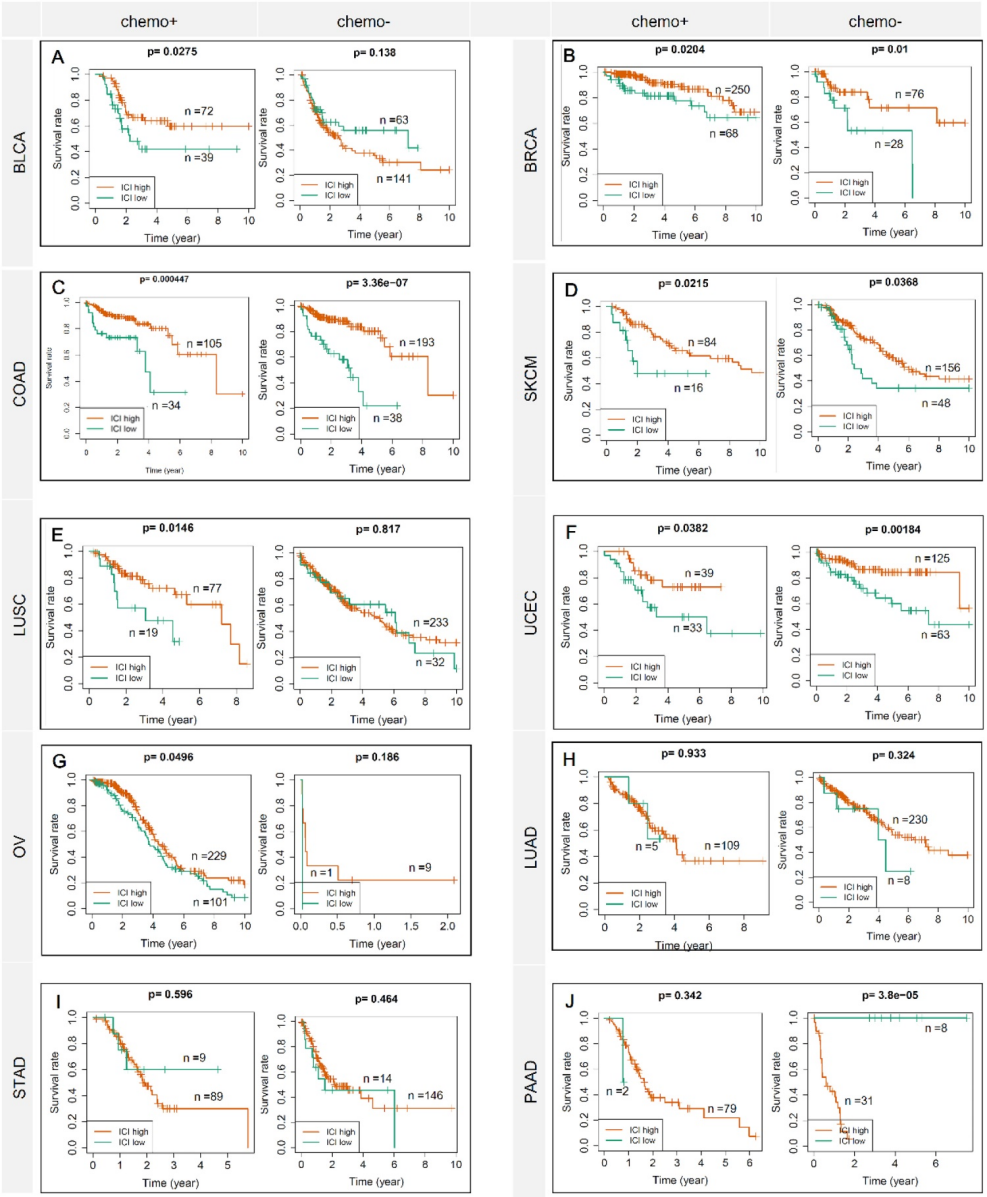
** ICI index indicates chemotherapy benefit in multiple cancers.** Each row represents a type of cancer. Each column represents a different grouping. A Kaplan-Meier Plotter was used to show the survival prediction ability of ICI index in multiple cancers (BLCA, BRCA, COAD, SKCM, LUSC, UCEC, OV, LUAD, STAD and PAAD). chemo+: chemotherapy patients; chemo-: non-chemotherapy patients.

Figure 1 showed the flow chart of data screening and analysis process. We first exclude the influence of radiotherapy on the prognosis of cancer patients (Table S1). Then among the remaining patients of pan-cancer, in order to ensure enough chemotherapy patients for study, we selected cancers which has chemotherapy patients >100 and chemotherapy patients >30% of total patients, including bladder urothelial carcinoma (BLCA), breast invasive carcinoma (BRCA), colon adenocarcinoma (COAD), lung adenocarcinoma (LUAD), lung squamous cell carcinoma (LUSC), ovarian cancer (OV), pancreatic adenocarcinoma (PAAD), skin cutaneous melanoma (SKCM), stomach adenocarcinoma (STAD) and endometrial carcinoma (UCEC) (Figure 2A-C). And the detailed clinic parameters of enrolled patients of 10 cancers were shown in Table S1. In our study, we divided patients into four groups according to the ICI index (high or low) and with or without chemotherapy (chemo+/-) (Table S2). We found that in 7 cancers of BLCA (P =0.0275), BRCA (P =0.0204), COAD (P <0.001), LUSC (P =0.0146), OV (P =0.0496), SKCM (P =0.0215) and UCEC (P =0.0382), high ICI index indicated good chemotherapy benefit (Figure 3A-G). And in 4 cancers of BRCA (P =0.01), COAD (P <0.001), SKCM (P =0.0368) and UCEC (P =0.00184), ICI index was also positively related with the prognosis of non-chemotherapy patients (Figure 3B-D, F). These results suggested that ICI index could indicate the outcome of cancer patients, especially in chemotherapy patients.

### The components and interaction of TIICs are diverse among different cancers

**Figure 4:**
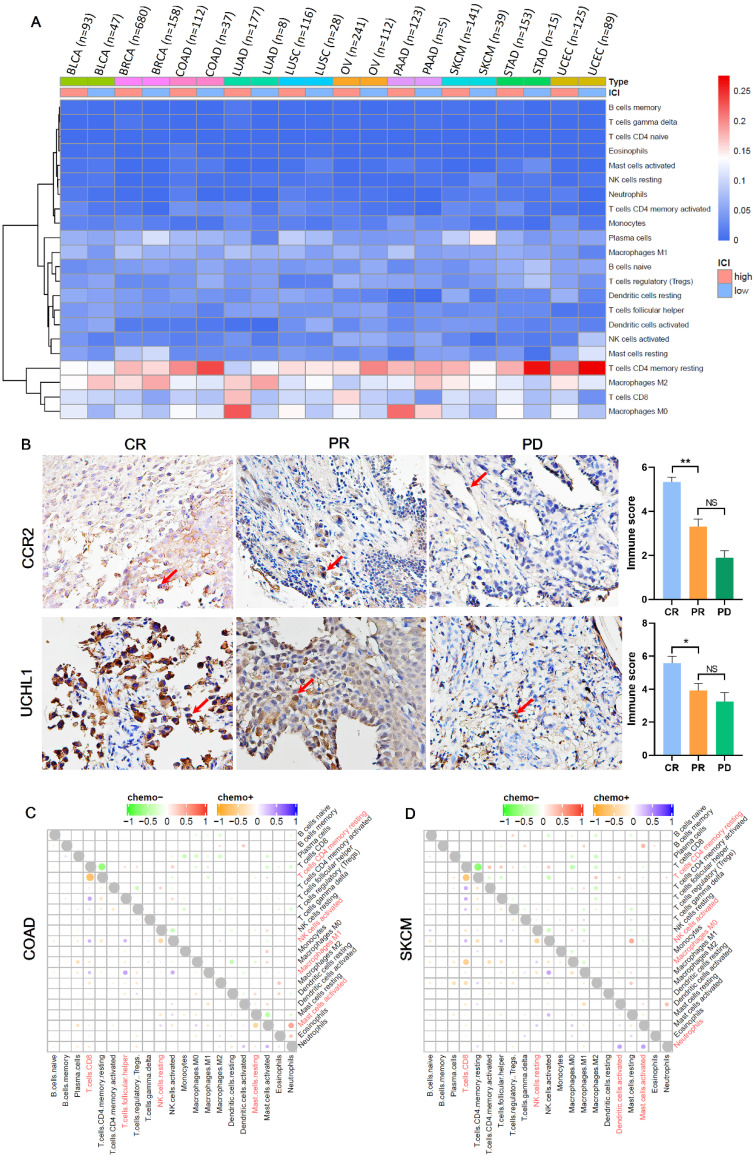
** The composition and interaction of TIICs are diversity among different cancers.** (**A**) Heat map showed the composition of TIICs in chemotherapy patients among cancer. (**B**) The expressions of Macrophage M0 (marker gene: CCR2) and CD8+ T cells (marker gene: UCHL1) in the preoperative puncture samples of NSCLC chemotherapy and the corresponding histogram of immunostaining score. CR: complete response; PR: partial response; PD: progressive disease. (**C, D**) The interaction between TIICs in chemotherapy and non-chemotherapy patients of COAD and SKCM.

Immune infiltrations are determined by the type and fraction of TIICs in TIME, and play an important role in inhibiting or promoting cancer growth 27. Figure 4A showed the components of TIICs in multiple cancers. We could see that the fraction of CD8+ T cells and macrophages M0 was high in chemotherapy patients with high ICI index, while the fraction of CD4+ memory resting T cells and macrophages M2 was low in chemotherapy patients with low ICI index. And based on the immunohistochemistry, we also found that compared with PR group, CR group has more infiltrations of macrophages M0 (marker gene: CCR2) and CD8+ T cells (marker gene: UCHL1) (Figure 4B; P_CCR2_ =0.035, P_UCHL1_ =0.006), which was consistent with the result of heat map. At the same time, we analyzed the interaction among TIICs in chemotherapy and non-chemotherapy patients. We found that in chemotherapy patients of COAD, SKCM, BLCA, LUSC, UCEC, LUAD and STAD, both CD8+ T cells and CD4+ memory resting T cells showed a strong negative correlation (Figure 4C, D; Figure S1A, C, D, F and G). And, in COAD, SKCM, BLCA, BRCA, UCEC, OV, LUAD and PAAD, compared with non-chemotherapy patients, the interaction between TIICs in chemotherapy patients was more abundant (Figure 4C, D; Figure S1A, B, D-F and H).

### Identifies six co-owned chemotherapy associated immune feature genes through correlation and causal network analysis

**Figure 5:**
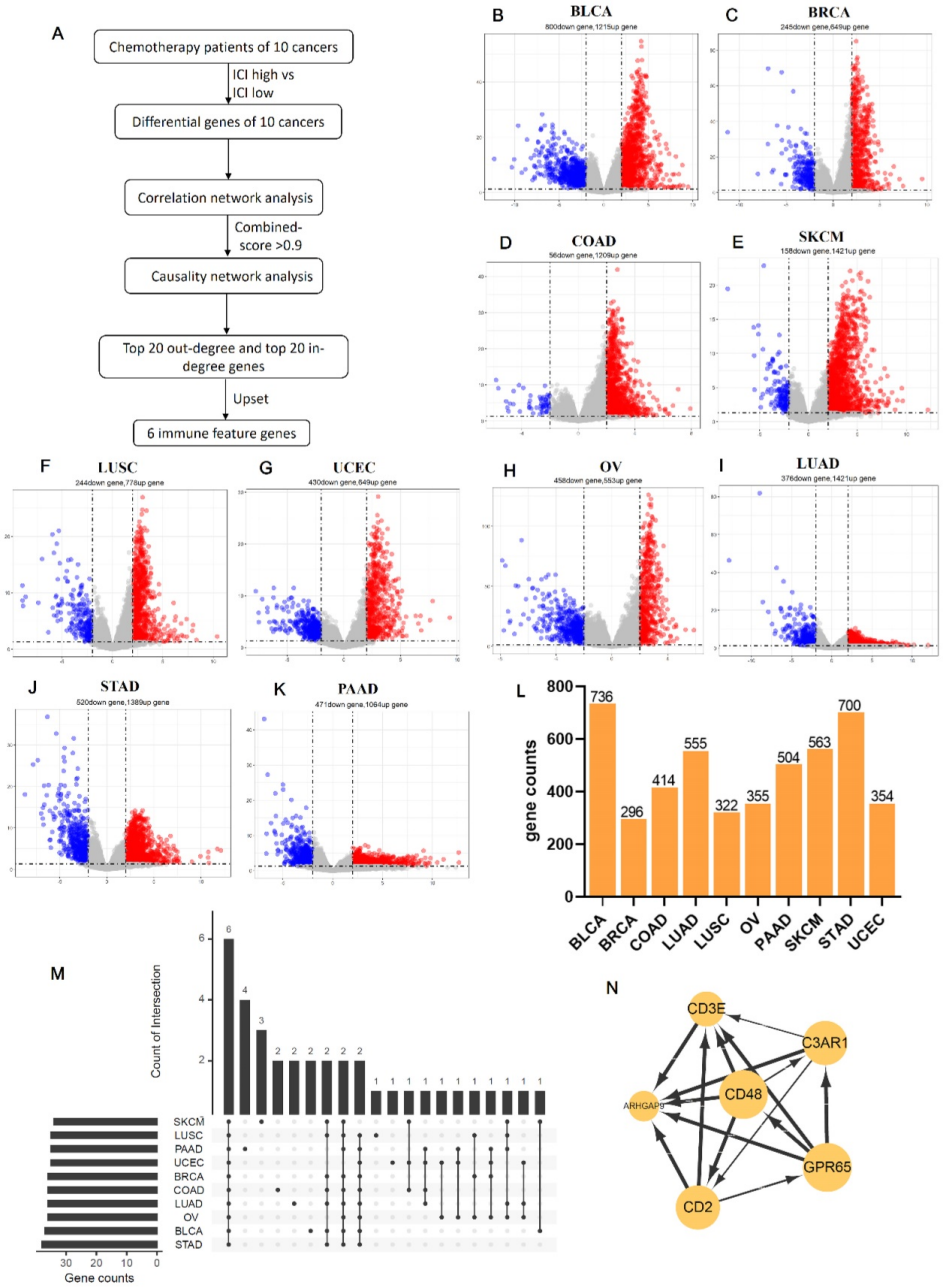
** Identifies six co-owned chemotherapy associated immune feature genes through correlation and causal network analysis.** (**A**) The flow chart showed how we determined chemotherapy associated immune feature genes by correlation and causal network analysis. (**B-K**) The volcano showed differential immune feature genes of 10 cancers. Blue indicates down-regulated genes, and red indicates up-regulated genes. (**L**) The histogram showed the number of genes screened by the combined score >0.9 after correlation network analysis of differential immune feature genes. (**M**) Upset displayed top 20 out-degree and top 20 in-degree genes obtained after causal network analysis. (**N**) The network relationship of chemotherapy associated immune feature genes in all chemotherapy patients.

Due to the prediction efficiency of ICI index is not fully confirmed so far, we analyzed which genes were related with chemotherapy benefit based on the transcriptome data (Figure 5A). In order to make the ICI group more credible, according to the value of ICI index, we selected the front 2/3 chemotherapy patients of high ICI group and the latter 2/3 chemotherapy patients of low ICI group to analyze differential immune feature genes (Figure 5B- K). Then, we calculated the correlation among these genes, and retained genes with combined score >0.9 (Figure 5L). Next, we selected top 60 immune feature genes based on the degree to execute the Bayesian causal network analysis, and drew these networks with weight >0.5 (Figure S2A-J). Finally, we extracted the top 20 in-degree and top 20 out-degree genes as the hub nodes of each cancer (Table 1) and identified six co-owned immune feature genes (CD48, GPR65, C3AR1, CD2, CD3E and ARHGAP9) in these cancers (Figure 5M). These genes were closely interacted in overall chemotherapy patients (Figure 5N).

### Construct a prediction model of chemotherapy benefit

**Figure 6:**
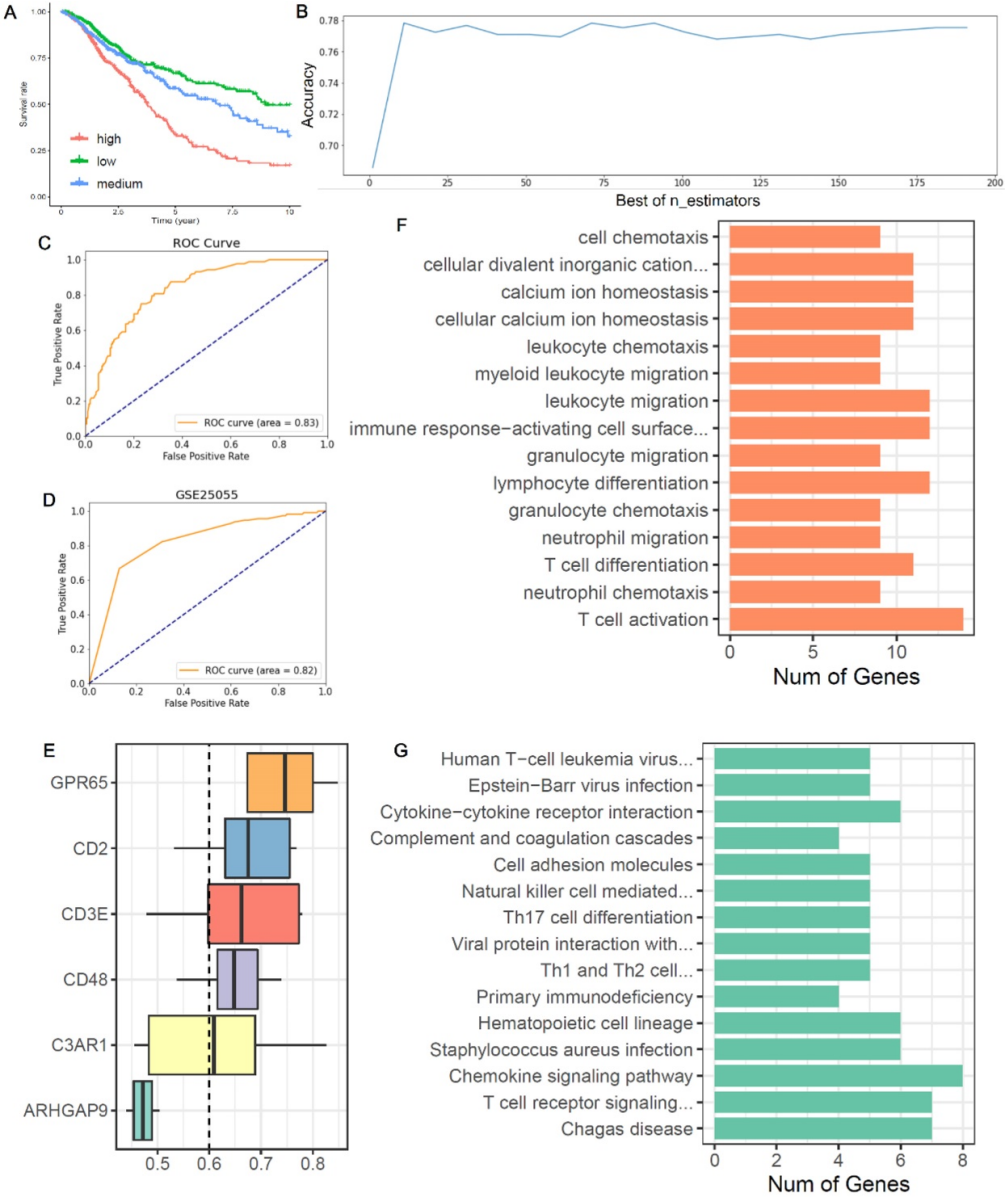
** Construction of a chemotherapy benefit prediction model and potential function analysis of immune feature genes.** (**A**) Based on six immune feature genes to construct a multivariate cox model, chemotherapy patients were divided into three groups according to the risk score. (**B**) The learning curve of n_estimators from 0 to 200. (**C**) ROC curve of this model in the test set. (**D**) ROC curve of this model in validation sets of GSE25055. (**E**) Boxplots show the functional similarity of six immune feature genes. The line in the box represents the average value of functional similarity. Proteins with high average functional similarity (cut-off >0.6) are considered interacting proteins. The dashed line indicates the cut-off value. (**F, G**) Display of biological functions and signal pathways enriched by immune feature genes.

Based on the expression of six co-owned immune feature genes in chemotherapy patients, a regression model was first constructed with multivariate cox (Figure S3A), and these patients were divided into three groups (high, medium, and low) according to the value of risk score (Figure 6A). Then, we selected the high and low groups to construct the random forest classifier model. By drawing the learning curve of n_estimators (Figure 6B) and performing grid search, we finally got the combination of each parameter of this model as RandomForestClassifier(n_estimators=11, max_depth=19, max_features=2, min_samples_leaf=1, min_samples_split=2). The weight of six immune feature genes was: 0.14771103, 0.19725147, 0.2050475, 0.15374589, 0.13619434 and 0.1600497, and the AUC was 0.83 (Figure 6C). Then, we extracted 30% of the total patients to verify the stability of this model and the AUC of 10 cancers all above 0.67 (Figure S3B-K). In addition, compared AUC of ICI index, we found that this model has higher AUC values in predicting the chemotherapy benefit (Table S6). Finally, we used two GEO datasets to validate the accuracy of this model, and we found that the AUC of GSE25055 and GSE14814 were 0.82 and 0.59, respectively (Figure 6D; Figure S3L). In general, this model has good efficacy in predicting the benefit of chemotherapy.

### Potential functions of six co-owned immune feature genes

From the boxplot, we could see that there were five genes (CD48, GPR65, C3AR1, CD2 and CD3E) above the cutoff value, indicating that these genes performed similar biological functions (Figure 6E). Next, we analyzed the biological functions of these immune feature genes. We found that these genes were mainly enriched in the “leukocyte chemotaxis”, “myeloid leukocyte migration”, “lymphocyte differentiation”, “T cell differentiation” and “T cell activation” (Figure 6F). And KEGG pathway analysis found that these genes were mainly enriched in “cytokine-cytokine receptor interaction”, “Th17 cell differentiation”, “chemokine signaling pathway” and “T cell receptor signaling pathway” (Figure 6G).

## Discussion

Compared with other cancer treatments, chemotherapy has the advantages of non-invasive and good compliance in patients 28, but also brings great harm to the patient's body 29, 30. It is still inconclusive in clinical for which population of cancer patients could benefit from chemotherapy certainly. How to divide patients into benefit subset or non-benefit subset remains the wildly discussed issue in clinical treatment of cancer nowadays 31. Whether patients could benefit from chemotherapy or not, partly depend on the unique cancer immune microenvironment of themselves 32, 33, ICI index which arisen from ESTIMATE analyzing of cancer transcriptome data could serve as a biomarker for chemotherapy benefit prediction 13, 14. As considering the variety of immune infiltration cells and their differential responses to chemotherapy, the efficiency of ICI index in prediction of chemotherapy benefit remains to be discussed in depth. In our work, we extended the conclusion in BLCA, BRCA, COAD, SKCM, LUSC, UCEC and OV, that high ICI index could instruct chemotherapy benefit in 932 patients based on TCGA datasets (P <0.05). And we found that the components of TIICs in the high/low ICI index group of chemotherapy patients and their interaction in with/without chemotherapy patients were diverse among different cancers. It offered the evidence that ICI index was not exactly right for the benefit prediction of chemotherapy in cancer patients, since ICI index only reflected the whole immune cell infiltration state.

Recently, studies also suggested that specific TIIC not total TIICs could indicate the therapeutic benefit of chemotherapy 34. In our study, we found that the interaction among TIICs was more frequent in patients within chemotherapy as contrasted to patients without chemotherapy (Figure 4C, D; Figure S1A-H). While tight interaction between CD8+ T cells and CD4+ memory resting T cells after chemotherapy often indicated the extending survival in patients (Figure 4A). These findings bring us the new vision, that is, it may be possible to establish a new benefit prediction method for cancer chemotherapy through isolating chemotherapy associated immune features.

Network analysis is different from conventional difference analysis, which could help us to detect key node factors related to event occurrence from omics data. In this study, by performing correlation and causal network analysis on the cancer transcriptome data, we isolated 40 chemotherapy associated immune feature genes of each cancer (BLCA, BRCA, COAD, SKCM, LUSC, UCEC and OV), and identified six co-owned immune feature genes: CD48, GPR65, C3AR1, CD2, CD3E and ARHGAP9. These genes were mainly enriched in the processing of “leukocyte chemotaxis”, “myeloid leukocyte migration”, “lymphocyte differentiation”, “T cell differentiation” and “T cell activation”. This is corresponding to the existed opinion that lymphocyte activation after chemotherapy indicates good drug response and survival benefit in patients 35.

In construction of the chemotherapy benefit prediction model within the selected six co-owned immune feature genes, we employed random forest classifying method to correct the weight of each feature genes. Total 973 cancer patients were included in model training, and 293 cancer patients were included in model testing, the AUC is 0.83, which exhibited better performance than ICI index in the prediction of chemotherapy benefit in cancer patients (Table S6). The performance efficiency of our model was finally validated with two external datasets GSE25055 (breast cancer, n=310) and GSE14814 (non-small cell lung cancer, n=131). All of this guarantee our model can be used for effective prediction of chemotherapy benefit in multiple cancers.

## Conclusions

In this study, we confirmed that high ICI index was related with chemotherapy benefit in multiple cancers and exhibited that TIICs were diverse among different cancers as responding to chemotherapy. By executing correlation and causal network analysis on cancer transcriptome data, we isolated several chemotherapies associated immune feature genes which included six co-owned immune feature genes. A chemotherapy benefit prediction model was constructed within these six co-owned immune feature genes and its efficiency was validated in external datasets. In short, this work offers a novel model with a shrinking panel which has the potential to guide optimal chemotherapy in cancer.

## Supplementary Material

Supplementary table 1: Clinic parameters of enrolled patients of 10 cancers.

Supplementary table 2: Grouping of cancer patients.

Supplementary table 3: Correlation network of pan-cancer.

Supplementary table 4: Degree of correlation network in pan-cancer.

Supplementary table 5: Causal network gene pairs with weight 0.5 of 10 cancers.

Supplementary table 6: Value of AUC between ICI and 6 genes model.

## Figures and Tables

**Table 1:** Hub genes of in-degree and out-degree of 10 cancers

BLCA	CD48, GPR65, P2RY10, CD74, C1QB, CCR4, ITK, SPI1, TYROBP, C3AR1, CD2, CD3D, CD3E, FCER1G, GZMA, LCP2, PIK3CG, SH2D1A, CASR, CXCL12, CXCR3, ARTP, DOK2, P2, DOCK, OK CCR8, CD8A, CXCL1, CXCL9, ICAM3, C3, CCL5, CCR2, CD3G, FPR1, FPR2, HLA-DRB1
BRCA	DOCK2, ITK, PTPRC, CD3G, PIK3CG, CXCR3, CCL21, C3AR1, CCR8, ICAM3, SST, LCP2, CCR4, CD3D, CXCR5, CXCL10, CXCL9, GZMA, ARHGAP9, CD3E, SPI1, G CCL, DOK2, P2, RY13 C1QA, CCR1, CCR5, C1QB, CD48, FPR2, CD2, CXCL11, FCER1G, HLA-DRB1, TYROBP
COAD	DOCK2, PIK3CG, PTPRC, CCR2, C1QA, CD48, C3AR1, LCP2, RGS18, GPR65, SLA, CCR1, CD3D, CNR1, P2RY10, CCL5, CCR7, CD2, CXCR1, FCER1G, FPR1, CXACR5, SHROBP, SHROBP, CCR8, ITK, CXCL10, DOK2, TRAT1, C1QB, CCL21, CD3E, CXCL9, GZMA, ARHGAP9
LUAD	SPI1, C3AR1, CD48, ICAM3, PTPRC, C1QB, FPR2, TYROBP, DOCK2, GPR65, SLA, C1QA, CD2, CD3E, C1QC, CD74, DRD2, SH2D1A, TAGAP, ARHGAP9, GZCRMA, ITXK, CXCR5, ITXCL10, PIK3CG LCP2, P2RY13, DOK2, CXCL9, IL12RB1, CXCR3, FPR1, ADCY8, CCL21, CCL5, CD8A
LUSC	DOCK2, CD74, CD3E, CD48, CXCR3, PTPRC, RGS18, P2RY10, CCR5, GPR65, LCP2, PIK3CG, SPI1, CCR2, CD2, CD3D, CXCL11, DOK2, FPR2, GZMA, C5AR1, CCR8, IL12RB1, C1, CXCL9, FCER1G, C1QC, CCR1, SH2D1A, CCR4, ITK, P2RY13, TYROBP, ARHGAP9
OV	LCP2, CCR1, CCR5, CD3E, CXCR3, RGS1, C3AR1, CD48, CXCL11, ARHGAP9, CCL5, CD2, FCER1G, PTPRC, IL12RB1, SH2D1A, FPR1, TYROBP, C1QA, C1QC, FPR3, GPR65, P2RY13, ITK, SPI1, CCR4, CXCL9, DOCK2, TAGAP, CD3G, CD74, CCL13, CCL19
PAAD	LCP2, CCR1, CCR5, CD3E, CXCR3, RGS1, C3AR1, CD48, CXCL11, ARHGAP9, CCL5, CD2, FCER1G, PTPRC, IL12RB1, SH2D1A, FPR1, TYROBP, C1QA, C1QC, FPR3, GPR65, P2RY13, ITK, SPI1, CCR4, CXCL9, DOCK2, TAGAP, CD3G, CD74, CCL13, CCL19
SKCM	CCR7, CD3G, TRAT1, C3AR1, CCL5, LCK, LPAR5, CD3E, CD2, CXCL9, GZMA, P2RY10, PTPRC, BDKRB1, C1QA, C3, CCR1, CCR5, FCER1G, SPI1, ARHGCRAP9, DPR2, C1Q ICAM3, SH2D1A, CCL21, GPR65, TYROBP, P2RY13, CD48, CD8A, HLA-DRB1
STAD	CD48, CD3D, P2RY10, CCR7, CCR5, CD2, FPR2, RGS18, SST, TYROBP, CCR4, CXCL9, CXCR2, P2RY13, C3AR1, CASR, CXCL10, SPI1, CCR1, CD8A, PIK3CG, IL12 G1, CD3G, CXCR5, FCER1G, LCP2, C1QA, CD3E, CCL21, CCR2, DOCK2, DOK2, GPR65, PTPRC, SH2D1A, ARHGAP9
UCEC	SH2D1A, IL12RB1, CD2, TRAT1, CCL19, CXCR3, CXCR5, C1QA, CCR7, GPR65, C1QB, CCL5, CCR5, CD3D, CD3E, KNG1, LCP2, RGS18, CCL21, CCR2, CXCL9, FCER1, DOCK2, RGS1, CD48, SLA, SPI1, TAGAP, C3AR1, CD8A, ICAM3, ARHGAP9, CCL13
